# Beyond the Narrowing: Atrial Fibrillation in Aortic Coarctation

**DOI:** 10.1016/j.cjcpc.2024.09.004

**Published:** 2024-09-21

**Authors:** Marie-Hélène Gagnon, Paul Khairy, Martin Aguilar

**Affiliations:** a Division of Pediatric Cardiology, University Hospital Center Sainte-Justine, Université de Montréal, Montreal, Québec, Canada; b Department of Medicine and Research Center, Montreal Heart Institute and Université de Montréal, Montreal, Québec, Canada

Individuals with congenital heart disease (CHD) represent a relatively small proportion of the general population in comparison with their disproportionate burden of atrial arrhythmias. Indeed, atrial arrhythmias are the most common cause of hospitalizations in adults with CHD ^1^ and are a major source of impaired quality of life. As patients with CHD age, the prevalence of atrial fibrillation (AF) increases to eventually surpass other forms of atrial arrhythmias and become the most common presenting arrhythmia.^2^ AF is associated with an increased risk of stroke and heart failure and poses a substantial burden on the health care system. In addition, in patients with CHD, the loss of atrioventricular synchrony or rapid ventricular response rates may be poorly tolerated and lead to haemodynamic compromise and occasionally cardiac arrest.

Coarctation of the aorta is a relatively common form of CHD, accounting for up to 8% of congenital defects. It is characterized by narrowing of the aorta near the ligamentum arteriosum, just beyond the subclavian artery. It carries a grim prognosis if not adequately identified and repaired. Advances in surgical repair and percutaneous stenting techniques allow for earlier intervention associated with improved patient outcomes.^3^ Nevertheless, large series have reported lower survival rates in patients with coarctation of the aorta than matched controls, even after successful repair.^4^ Hypertension, left ventricular outflow tract obstruction, aortic valve regurgitation, recoarctation, and other aortopathies are among the complications reported in patients with repaired coarctation.^5^ AF has also been reported, but its incidence, predictors, and prognostic significance are not well defined.

## Repaired Coarctation of the Aorta and Atrial Fibrillation

In this issue of the *Canadian Journal of Cardiology—Pediatric & Congenital Heart Disease*, Egbe et al.^6^ report on the incidence, risk factors, and prognostic significance of new-onset AF in a retrospective cohort of 782 patients with repaired coarctation of the aorta followed for a mean of 6.2 years. The median age at repair was 3 years, and the median age at first encounter for the study was 32 years, with a relatively well-balanced ratio of males to females. Most patients (32%) were treated with end-to-end anastomosis, and only a minority (6%) had percutaneous stenting of the coarctation. As expected, more than half (52%) of the study participants carried a diagnosis of hypertension on inclusion in the cohort. Principal findings were that (1) the incidence of AF at follow-up was 0.9% per year; (2) age, hypertension, left atrial dysfunction, and left ventricular hypertrophy were predictors of new-onset AF on multivariable analysis; and (3) new-onset AF was associated with adverse cardiovascular events (heart failure hospitalization and all-cause mortality). Most patients (26 of 42; 62%) with new-onset AF required cardioversion and antiarrhythmic drug therapy, 17% (7 of 42) underwent catheter ablation, and 69% (29 of 42) were anticoagulated (vitamin K antagonists or direct oral anticoagulants).

The authors should be commended for contributing the most extensive series addressing the incidence and predictors of new-onset AF in patients with repaired coarctation of the aorta. With the increasing number of adults with CHD reaching well into adulthood, it is becoming increasingly important to characterize the spectrum of long-term complications from their cardiac defect and corresponding correction/repair. The accompanying paper makes the vital point that patients with repaired coarctation of the aorta are at significant risk, not only of developing AF but of the arrhythmia manifesting at a much younger age than in the general population. These results are consistent with recent reports suggesting that, on average, AF in patients with CHD occurs 30 years earlier than in the general population.^7^ Hence, even though the reported incidence is “only” 0.9% per year, the implications of being diagnosed with AF in the third decade of life are potentially highly significant.

## Feeling the Pressure

The authors identified several predictors of new-onset AF in adults with repaired coarctation of the aorta (age, hypertension, left-atrial reservoir strain, and left ventricular mass index). This sheds some light and invites several interesting questions about the mechanisms of AF in this patient population. It appears likely that the pressure overload posed by the coarctation onto the left ventricle and, consequently, the left atrium could facilitate profibrillatory left atrial structural remodelling. The instrumentation of the atria during surgical repair in selected patients requiring cardiopulmonary bypass is also likely to promote the occurrence of atrial arrhythmias, especially scar-mediated reentry, which could serve as a “trigger” for AF.

It is interesting to note that a substantial (52%) proportion of patients carried a diagnosis of hypertension at the time of inclusion in the cohort. Hypertension is a well-described occurrence in patients with repaired coarctation and a known risk factor for AF in the general population. These observations and the finding that the left ventricular mass index was also associated with new-onset AF support the pressure overload hypothesis as a crucial pathophysiological driver of AF in this patient population. Proactive screening and early treatment of hypertension are, therefore, of utmost importance. Finally, Egbe et al. also reported new-onset AF to be associated with heart failure hospitalization and/or all-cause mortality, highlighting the clinical significance of AF on patient prognosis.

## Monitoring for Atrial Fibrillation: You Only Find What You Are Looking For

A noteworthy limitation of the present study is the lack of a standardized rhythm monitoring protocol. In fact, there was an average of only 4 electrograms per patient and 0.3 Holter monitor per patient over >6 years of median follow-up. Given that AF is often paroxysmal and/or asymptomatic, such low-density rhythm monitoring that reflects standard clinical practice is almost certain to underestimate the true incidence of AF by a wide margin. The importance of how patients are monitored on the reported (or apparent) incidence of AF is increasingly being recognized. As an illustrative example from a different setting, the 12-month arrhythmia-free survival in adults undergoing catheter ablation for AF decreases from 92.5%, in patients monitored with three 24-hour Holter monitors, to 52.6%, in the same patients if continuously monitored with an implantable cardiac monitor.^8^ In other words, the rate of AF detection is *massively* dependent on how intensely patients are monitored. Consequently, the reported incidence of 0.9% per year in the present study is undoubtedly a “lower bound” on the actual incidence of AF in this patient population. The true incidence of AF is likely to be significantly higher.

This poses an interesting and perplexing clinical question: should patients with repaired coarctation of the aorta be systematically screened for atrial arrhythmias/AF? If the incidence of AF is (probably much higher than) 0.9% per year and new-onset AF is associated with worse cardiovascular outcomes, early detection may allow early intervention, risk factor modification, and improved outcomes. The cost-effectiveness of such an approach remains to be determined.

## Back to the Future

Paediatric cardiology is a rapidly evolving field, as exemplified by advances in the management and repair techniques/tools for children with coarctation of the aorta.^9^ The timing for coarctation repair remains a moving target, balancing the benefits of early intervention with the risk of requiring reoperation later in life, among many other considerations. Brown et al.^3^ have reported that the proportion of patients treated with end-to-end anastomosis, the classic “gold standard” repair, decreased from >90% before the 1950s to approximately 40% in patients treated after the year 2000. Over the same period, alternative surgical approaches (subclavian flap and grafts) and percutaneous ballooning and stenting techniques have been developed and gained significant ground. Therefore, the results reported in the present study should be considered in the context of the evolving clinical practice in patients with coarctation. Although the study did not explicitly report the date range for the coarctation repair, the inclusion criteria suggest that most interventions were performed in the 1990s. Clinicians should remain alert to the evolving practice in children with coarctation, which will likely impact the incidence, risk factors, and prognostic implications of long-term complications in general and as they relate to atrial arrhythmias.

## Future Directions

Patients with CHD are often excluded from AF catheter ablation and anticoagulation trials or are under-represented such that there is a paucity of evidence to guide clinical management. In Egbe et al.’s study, many patients required cardioversion, antiarrhythmic drugs, and/or catheter ablation. How these patients respond to these treatments remains to be investigated. It would be interesting to learn more about the intracardiac findings and arrhythmia outcomes in the subset of patients who underwent catheter ablation—did they have a higher prevalence of nonpulmonary vein triggers? Were there macro-reentrant circuits?

Along the same lines, the benefit of anticoagulation in these patients has yet to be formally demonstrated. Coarctation of the aorta is considered “moderate” along the spectrum of CHD complexity. Expert consensus-based recommendations state that long-term anticoagulation is reasonable in patients with moderate forms of CHD who have sustained AF or intra-atrial re-entrant tachycardia.^10^ Moreover, the high prevalence of hypertension in patients with repaired coarctation is such that a significant proportion of patients with AF meet the standard guideline-directed indication for anticoagulation. On the other hand, there may be a subset of young and active patients without hypertension in whom the benefits of lifelong anticoagulation do not outweigh the risks. Further data are required to guide clinical decision-making.

A variable that has not been formally assessed is the impact of the timing of intervention on the risk of AF. There is evidence that earlier intervention may be associated with lower rates of hypertension after repair.^11^ If systemic hypertension-mediated chronic pressure overload is an important pathophysiological mechanism of AF in these patients, one would expect earlier intervention to reduce the risk of AF, along with other hypertension-related complications.

In conclusion, this study provides critical new insights into the incidence, risk factors, and prognostic significance of new-onset AF in patients with repaired coarctation of the aorta. Many fascinating and highly clinically relevant questions remain to be answered in this patient population, such as the role of systematic rhythm monitoring for AF and how to best manage young patients with a history of coarctation who develop AF, in view of optimizing patient outcomes.
